# Removal of Superior Maxilla

**Published:** 1873-08

**Authors:** 


### Removal of Superior Maxilla.

Dr. J. J. CHISHOLM in the *Medical Record* says of the best method of removing the upper jaw:

Having for many years taught and practiced Dieffenbach's operation for the removal of the upper jaw as the best method, both as to the ready exposure of the entire bone, and leaving the least deformity as a permanent result, I have been much surprised to find so little mention of this excellent method. Chelius, in his system of surgery, refers to Dieffenbach's method in one line only. In the more recent works of Holmes, Erichsen, Furgerson, Gross, Gant, and others, the operation appears to have been completely lost sight of. Dieffenbach's operation consists in making the incision in the median line of the face, commencing at the root of the nose; a short incision joining the first at right angles, extends from the root of the nose to the inner angle of the eye. The lower lid being drawn downwards, the knife is carried along the entire length of the conjunctival cul-de-sac, separating this lid from its orbital connection, and utilizing the entire length of the lower lid in the horizontal flap. When the flap, as defined by the vertical and horizontal incision, is dissected up, it will lay bare the entire front, and if necessary, side of the face, without having divided any large vessel, or any important nerve branch. With such an exposure, the superior maxillary bone can be isolated with great ease, as every surface of contact with neighboring bones can be clearly brought into view. With no additional incision, I found no difficulty in removing from the living subject the superior maxilla, malar, and palate bone, which enabled me to extirpate a large fibroid, with extensive adhesions to the roof of the pharynx. After the removal of the maxilla, when the flap is brought back to its normal situation, and carefully adjusted by several points of suture, union speedily ensues. This operation leaves so little deformity that, in the majority of cases, the line of incision will escape detection, unless the scar be sought. The inferior lid retains all of its movements. The sides of the face present their normal appearances, unscarred, with nerves and vessels intact. The eye muscles can be so nicely manipulated through this bold flap, that all the movements are retained. As the suspensory ligaments of the eye ball remain adherent to the roof and sides of the orbit, there is no drooping of the eye ball. Immediately after the suture are placed, I have seen the patient move the eyes in parallel axis, showing perfect control.

